# MINING: EPA Tackles Fracking

**DOI:** 10.1289/ehp.118-a199

**Published:** 2010-05

**Authors:** John Manuel

**Affiliations:** **John Manuel** of Durham, NC, is a regular contributor to *EHP* and the author of *The Natural Traveler Along North Carolina’s Coast* and *The Canoeist.*

With the push for energy independence and fuels that emit fewer greenhouse gases, domestically produced natural gas has been growing in popularity. But alongside this growth have come concerns that hydraulic fracturing (“fracking”), a procedure used in the extraction of natural gas and oil, may pollute ground and surface waters. Responding to increasing public pressure for federal action and a call by the U.S. House of Representatives Appropriations Conference Committee, the U.S. Environmental Protection Agency (EPA) announced 18 March 2010 it will conduct a comprehensive study to investigate the potential adverse effects of fracking on water quality and public health.

Natural gas provides almost 25% of the U.S. energy supply and could provide 50% by 2035, according to the 2010 report *Fueling North America’s Energy Future* by consultancy IHS Cambridge Energy Research Associates. In recent years, vast new deposits of natural gas have been discovered in layers of shale thousands of feet underground. Some of these deposits, such as the Marcellus Shale running under the Appalachian Basin, lie beneath watersheds supplying drinking water to millions of people. In many locations fracking—in which a mixture of water, sand, and chemicals is injected into natural gas wells under high pressure—occurs within hundreds of feet of residences that use wells for drinking water.

Recent evidence suggests fracking may have contributed to groundwater contamination with methane in some instances and that proprietary chemicals used in the procedure could theoretically pose a public health threat. However, because groundwater supplies and natural gas deposits are often separated by thousands of feet of rock and earth, and groundwater can be contaminated by many sources, it is difficult to establish a definitive connection between contaminated drinking water and fracking. Further, there has been very little in-depth research on the subject with respect to drilling in shale beds.

In 2005 Congress exempted fracking from regulation under the Safe Drinking Water Act partly on the basis of the EPA report *Evaluation of Impacts to Underground Sources of Drinking Water by Hydraulic Fracturing of Coalbed Methane Reservoirs*. The authors of this report wrote that hydraulic fracturing poses “minimal threat” to drinking water and that “additional or further study is not warranted at this time.” However, the study involved no direct monitoring of water wells but instead relied on existing peer-reviewed literature and interviews with industry and state and local government officials. It also was strictly limited to one specific type of drilling and did not address the effects in substrates other than coalbeds.

Operators surround drill holes with steel casings cemented into place to prevent groundwater contamination during fracking. In addition, a large volume of “back-flow fluids,” on the order of hundreds of thousands of gallons per well, are brought to the surface during drilling and production. Back-flow fluids are typically stored in on-site pits (which, depending on state regulations, may or may not be lined) and ultimately are disposed of either by injection into EPA-approved underground wells or by delivery to municipal waste treatment facilities. In January 2010 state officials testified before the Ohio House of Representatives that standards and enforcement regarding oil and gas well construction are not always adequate to ensure proper performance.

The EPA has reallocated $1.9 million for the new study in this fiscal year and will request further funding in the President’s FY2011 budget proposal. The agency’s scoping document identifies three major categories for research: characterization of the fracking life cycle, potential relationships to drinking water sources, and potential health and environmental hazards. Explicit research goals, as yet undefined, will be divided into short term (1–3 years) and long term (3–5 years).

“We’re very pleased that EPA is doing this study,” says Amy Mall, a senior policy analyst with the Natural Resources Defense Council (NRDC), which has long pressured Congress for federal regulation of fracking. “There are communities around the country that are very concerned because their water has been contaminated or could be contaminated.”

The NRDC also supports passage of the Fracturing Responsibility and Awareness of Chemicals (FRAC) Act, which was introduced in Congress in 2009. The FRAC Act would permit regulation of hydraulic fracturing under the SDWA and would require oil and gas companies to disclose the chemicals used in fracking operations.

Energy In Depth, a nonprofit organization representing the oil and gas industry, has lobbied against the FRAC Act, but supports the new EPA study in concept. “We’ve said from the outset that any updated study in this area should be science-based, peer-reviewed, and completely isolated from political design or interference,” says Chris Tucker, spokesman for Energy In Depth. “Assuming those criteria are met, we’re confident the new study will end up reaching the same conclusions that were produced by the old study.”

## Figures and Tables

**Figure:**
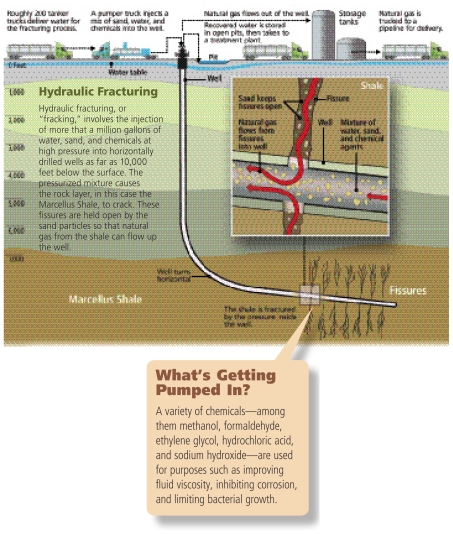
What’s Getting Pumped In? A variety of chemicals—among them methanol, formaldehyde, ethylene glycol, hydrochloric acid, and sodium hydroxide—are used for purposes such as improving fluid viscosity, inhibiting corrosion, and limiting bacterial growth.

